# Defect passivation of hafnium oxide ferroelectric tunnel junction using forming gas annealing for neuromorphic applications

**DOI:** 10.1186/s40580-025-00481-6

**Published:** 2025-03-24

**Authors:** Manh-Cuong Nguyen, Kyung Kyu Min, Wonjun Shin, Jiyong Yim, Rino Choi, Daewoong Kwon

**Affiliations:** 1 Program in Semiconductor Convergence and 3-D Convergence Center, Department of Materials Science and Engineering, Inha University, Incheon, 22212 South Korea; 2 Department Inter-University Semiconductor Research Center, Department of Electrical and Computer Engineering, Seoul National University, Seoul, 08826 South Korea; 3 Department of Semiconductor Convergence Engineering, Sungkyunkwan University, Suwon, Gyeonggi-Do 16419 South Korea; 4 Department of Electronic Engineering, Hanyang University, Seoul, 04763 Korea

**Keywords:** Ferroelectric, Tunnel junction, Hafnium oxide, Synaptic device, Neuromorphic, Defect passivation, MFIS, Trap-assisted tunneling

## Abstract

Forming gas annealing (FGA) is applied to HfO_x_ ferroelectric tunnel junction (FTJ) synaptic devices to passivate defects and reduce trap-assisted-tunneling (TAT). Without FGA, TAT caused by defects in metal–ferroelectric–insulator–semiconductor (MFIS) FTJ stack dominates the conduction mechanism in FTJs and results in no memory window (MW). The reduction of defects or TAT after FGA reveals the effect of polarization switching on the FTJ performance. Consequently, linear/symmetric potentiation and depression (P/D) characteristics of FTJ after FGA with stable repeatability are obtained. Owing to the FGA-induced linearity and symmetricity of P/D, a learning accuracy of approximately 90% is achieved via pattern recognition simulations utilizing HfO_x_ FTJ crossbar.

**Graphical Abstract:**

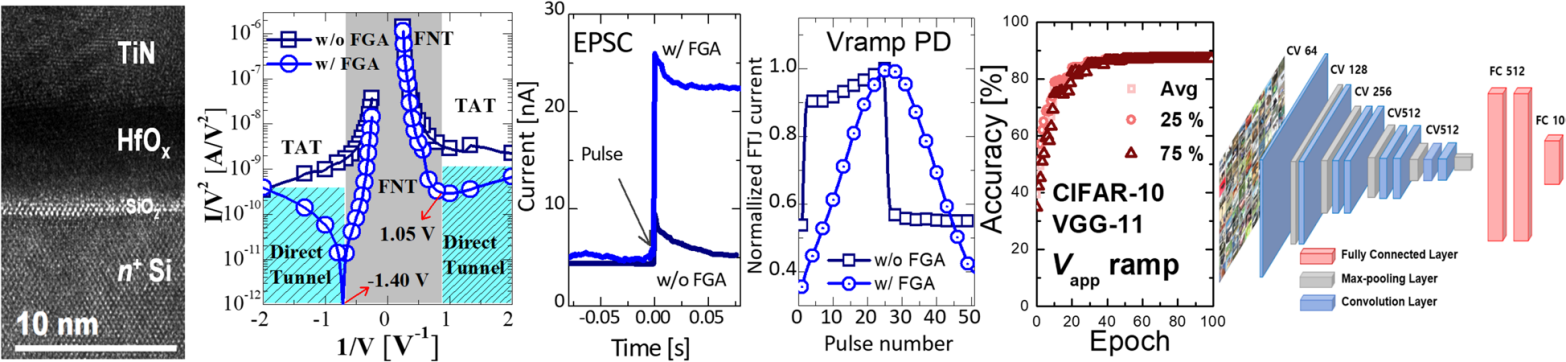

## Introduction

Owning to multi-state nonvolatile memory operations, robust retention, and endurance, ferroelectric synaptic devices for neuromorphic computing have been investigated [1–6]. Specifically, HfO_x_-based ferroelectric field-effect transistors and ferroelectric tunnel junctions have attracted significant attention owing to their perfect compatibility with complimentary metal–oxide–semiconductor fabrication processes, excellent ferroelectricity with nanometer thickness, stable multi-state memory, and symmetric potentiation and depression characteristics [7–10].

HfO_x_-based FTJs are suitable for low-power and high-density neuromorphic device arrays because of their cross-point structure and high resistance caused by the low tunneling current regulated by polarization and the unidirectional tunneling current flow [4–6, 9]. Several structures, such as metal–ferroelectric–metal (MFM), metal–ferroelectric (FE)–insulator (IL)–metal (MFIM), and metal–ferroelectric–insulator–semiconductor (MFIS), have been proposed for the implementation of these FTJs. In particular, FTJs with MFIS and MFIM structures have been highlighted because of their wide memory windows [11].

HfO_x_-based ferroelectric materials, particularly doped HfO_x_, have attracted considerable attention owing to their compatibility with Si, nanoscale ferroelectric properties, high endurance, and robust retention. In comparison to metallic ion-doped HfO_x_ (such as Al, Si, and La) [12–15], laminated Hf–Zr–O (HZO) is considered an attractive candidate owing to its wide doping range and process window for ferroelectricity formation [16]. Compared with HZO, the ferroelectricity of undoped HfO_x_ begins to appear at high temperatures (over 700 °C) [17]. This is advantageous considering the heat budget by the subsequent process steps after the ferroelectricity formation since HZO typically loses its ferroelectricity at high temperatures (higher than 600 °C). However, the ferroelectricity formation temperature of undoped HfO_x_ is limited by the leakage current increase (due to TAT), resulting in deteriorated ferroelectric properties of undoped HfO_x_ compared to those of HZO [17]. To reduce the effect of TAT on the FTJ performance, an insulator is added to reduce TAT at a low electric field (because the charge centroid shifts away from the interface) [17]. However, defects in ferroelectric devices in the MFIS and MFIM structures may cause severe degradation of memory properties and reliability [18]. Numerous methods, such as atomic layer deposition (ALD) for the reduction of metal-FE interface defects, fluorine passivation, and FGA, have been proposed for the passivation of defects in ferroelectric devices [18–20]. FGA is one of the most effective methods for improving the reliability and ferroelectricity of HfO_x_-based ferroelectric devices (using MFIS structure) and enhancing their ferroelectricity [20]. However, the effects of FGA on HfO_x_-based synaptic devices with MFIS structures have not been investigated. Therefore, elucidating the effects of interface defects on the performance of FTJs with IL is essential for minimizing degradation and stabilizing memory operations.

In this study, HfO_x_ MFIS FTJs were fabricated to investigate the effects of FGA on FTJs for synaptic applications. The effect of charge trapping was clarified by analyzing the conduction mechanism of the FTJs. Defect passivation utilizing FGA has been proposed to reduce TAT and improve the linearity and symmetry of the P/D. The effects of the improved P/D linearity and symmetry via FGA on the pattern recognition accuracy for the Modified National Institute of Standards and Technology (MNIST) data were determined. The applicability of FTJs as synaptic devices in a convolutional neural network (CNN) was simulated using the Canadian Institute for Advanced Research (CIFAR-10) dataset.

## Experimental

**Fig. 1:**
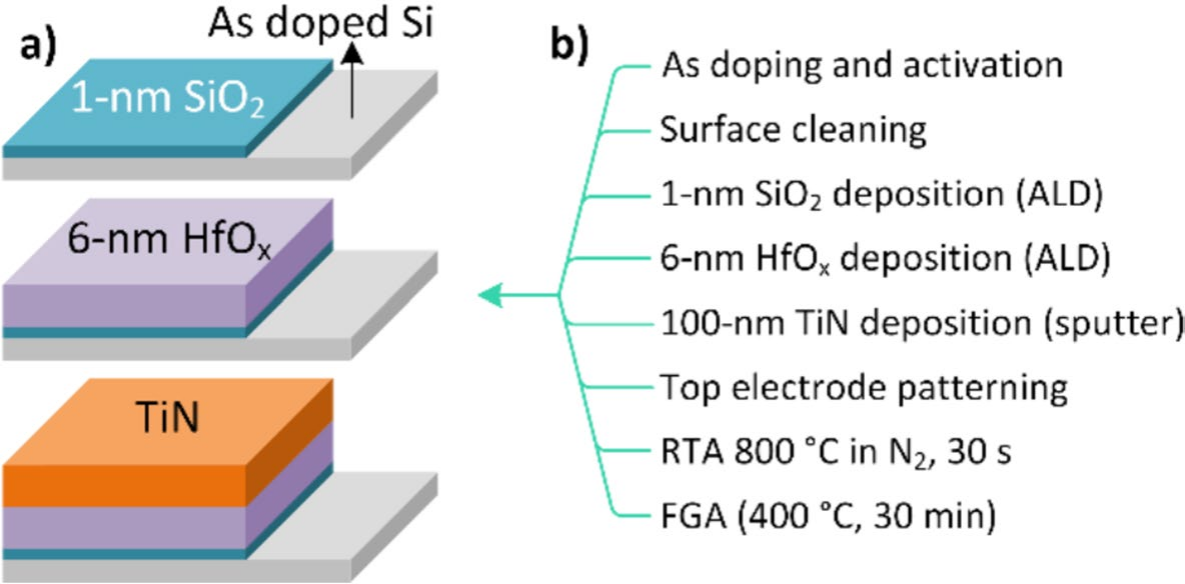
HfO_x_ MFIS FTJ fabrication illustration (**a**) and fabrication process flow (**b**)

Figure 1a, b illustrate the fabrication and process flow of the HfO_x_ FTJ. A Si wafer was doped using a dose of 5 × 10^15^ cm^−2^ (Arsenic, 40 keV), activated by rapid thermal annealing (RTA) at 950 °C in N_2_ ambient for 5 s. After a standard cleaning process and removal of the native oxide, 1- and 6-nm-thick SiO_2_ and HfO_x_ layers were sequentially deposited via ALD. Subsequently, 100 nm TiN was deposited as the top electrode via sputtering at room temperature. The MFIS devices were patterned over an area of 100 × 100 μm^2^. For the transition to the orthorhombic phase (o-phase), rapid thermal annealing (RTA; 800 °C, 30 s) was carried out as post-metal annealing (PMA) under N_2_ ambient conditions for the ferroelectricity of pure HfO_x_. However, RTA at high temperatures resulted in grain boundaries and defect formation at the interface of the MFIS FTJ. Therefore, hydrogen passivation was performed by forming gas annealing (FGA; 96% of N_2_ and 4% of H_2_) at 400 °C for 30 min to passivate the defects in the FTJ stack.

**Fig. 2:**
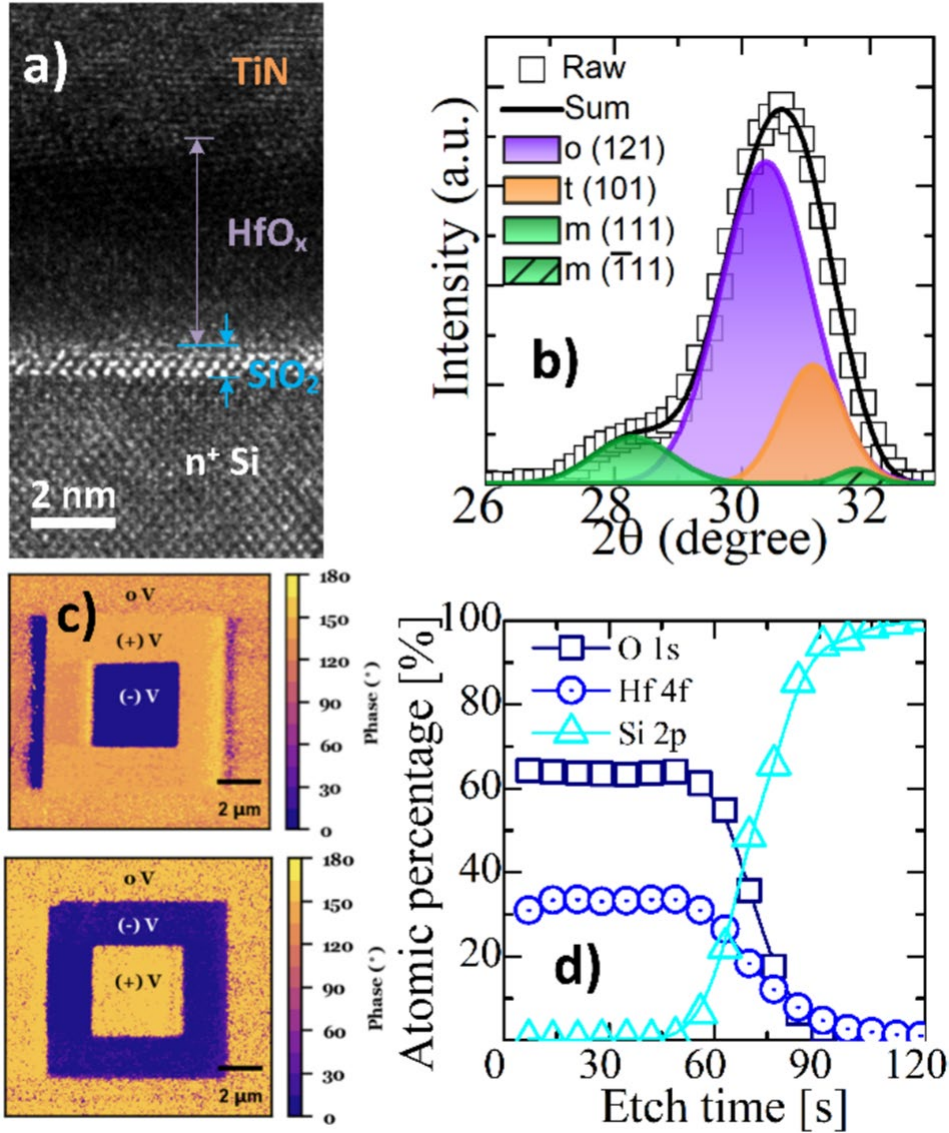
Cross-sectional TEM image of HfO_x_ MFIS FTJ (**a**), XRD spectra deconvolution of HfO_x_ film after RTA and TiN removing (**b**), phase-contrast PFM images at reverse bias (top) and forward bias (bottom) with opposite bipolar remanent polarization states at ±4 V (**c**), and XPS atomic depth profile crossing HfO_x_/SiO_2_/Si stack (**d**)

A cross-sectional transmission electron microscopy (TEM) image of the HfO_x_ FTJ, X-ray diffraction spectrum of HfO_x_ after RTA and TiN removal, phase-contrast piezoresponse force microscopy (PFM), and atomic percentage depth profile crossing HfO_x_/SiO_2_/Si using X-ray photoelectron spectroscopy (XPS) are shown in Fig. 2a–d, respectively. The crystallization of HfO_x_ is shown in Fig. 2a. The HfO_x_ film annealed at 800 °C was fabricated with mostly o-phase (Fig. 2b), which is also confirmed in our previous reports [18, 19]. The phase-contrast PFM images demonstrate stable, bipolar, and remnant polarization states that can be overwritten into the opposite polarization state (Fig. 2c). The elemental depth profile of the XPS crossing HfO_x_/SiO_2_/Si shows a continuously diffused interlayer between HfO_x_ and Si.

**Fig. 3:**
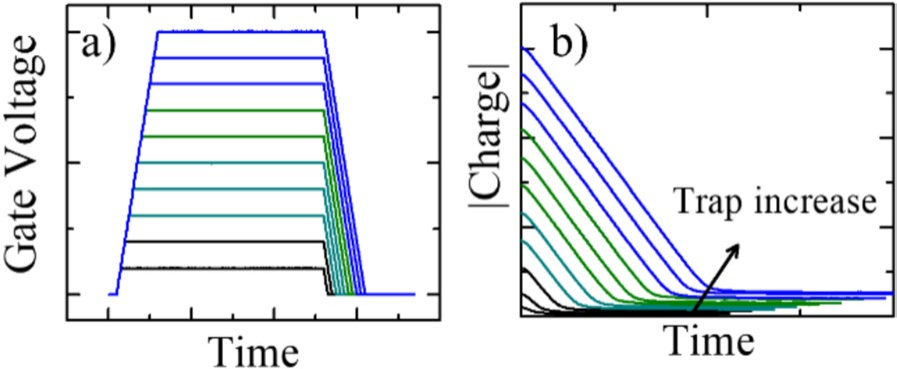
Illustration of constant ramping rate SPCP pulse configuration (**a**) and charge extraction at the end of falling edge of each pulse (**b**)

A single-pulse charge pumping (SPCP) method was used to measure the defect density (N_trap_) in the FTJ stacks before and after FGA. SPCP is an effective tool for extracting effective defect densities in MFM or MOS stacks [21]. In a SPCP measurement, only defects with capture time shorter than pulse width and emission time longer than the falling edge are detected. Different pulses with the same ramping rate were applied to the top electrode (Fig. 3a) to record the charging current passing through the bottom electrode (I_SPCP_). A constant ramp rate is used to make sure the emission time for each defect level (corresponding to each voltage level) be a constant (time from a certain voltage to V_base_ is unchanged among the measurement conditions). The total charge transferred through the bottom electrode was calculated by integrating I_SPCP_ with time. Subsequently, N_trap_ was extracted at the end of each pulse (illustrated in Fig. 3b) to calculate the effective density of the trapped charges remaining in the device:

1 $${N}_{trap}=\frac{{Q}_{SPCP}}{A*q}=\frac{\int {I}_{SPCP}dt}{A*q},$$

where *Q*_*SPCP*_, *A*, and *q* denote the remaining charges after the falling edge of the pulse, the FTJ area, and the elemental charge, respectively.

A Keithley 4200-SCS with a fast pulse measurement unit was used to characterize the polarization characteristics and perform SPCP measurements. A Keysight B1500 with a waveform generator/fast measurement unit B1530 was used to characterize the direct-current (DC) measurements, P/D, retention, and endurance characteristics.

## Results and discussion

**Fig. 4:**
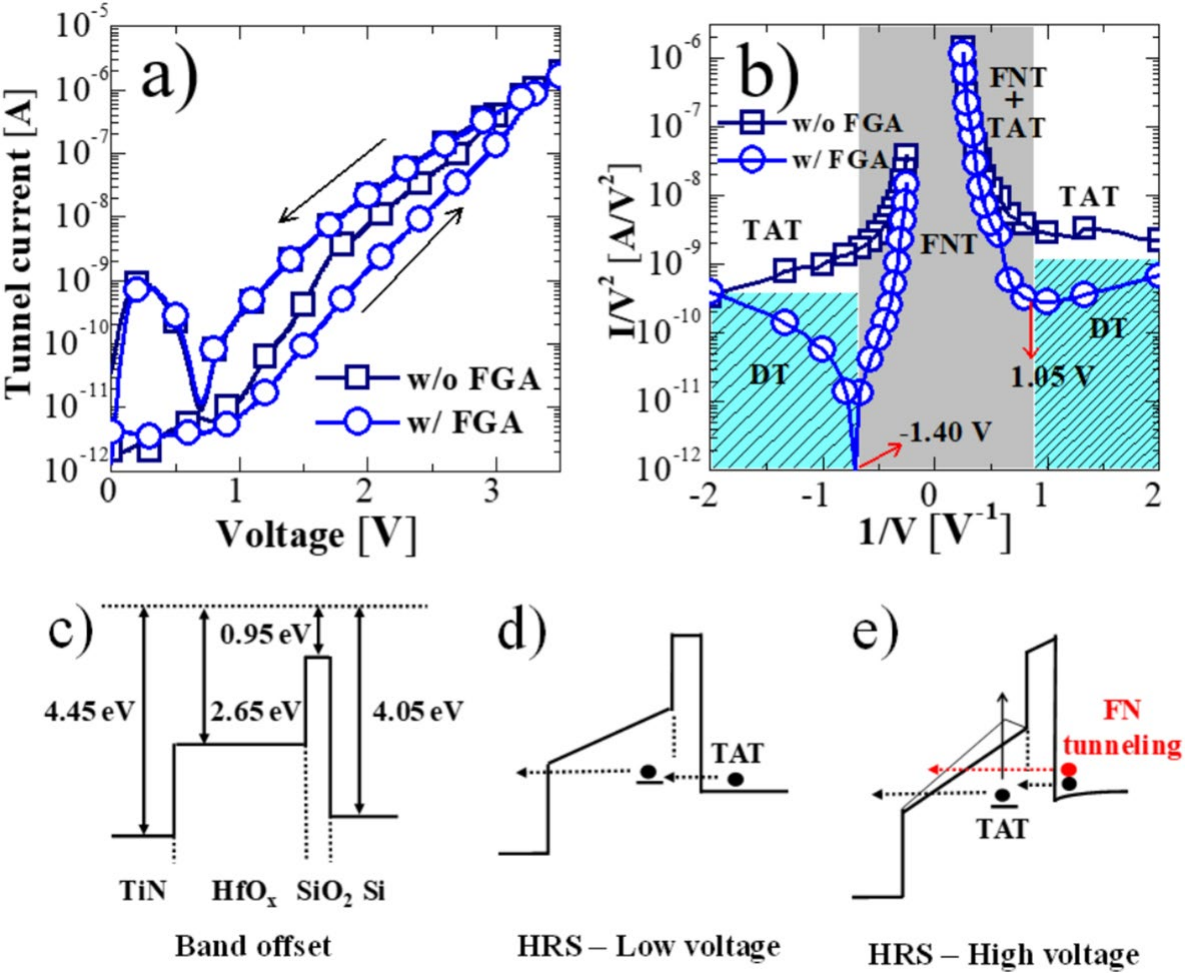
Hysteretic tunneling current–voltage characteristics (**a**), *I*_tun_/*V*_app_^2^ versus 1/*V*_app_ plots of forward sweeping for the FTJs before and after FGA (**b**), conduction band offset of the FTJ (**c**), schematics of the conduction mechanism of the FTJ in the HRS at low voltage (**d**) and high voltages (**e**)

**Fig. 5:**
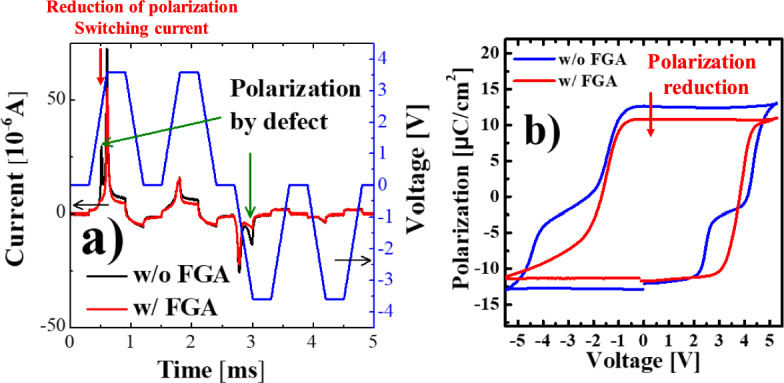
Schematic of PUND measurement and transient IV curves of the FTJs before and after forming gas annealing (**a**) and polarization characteristics of the pristine FTJs

**Fig. 6:**
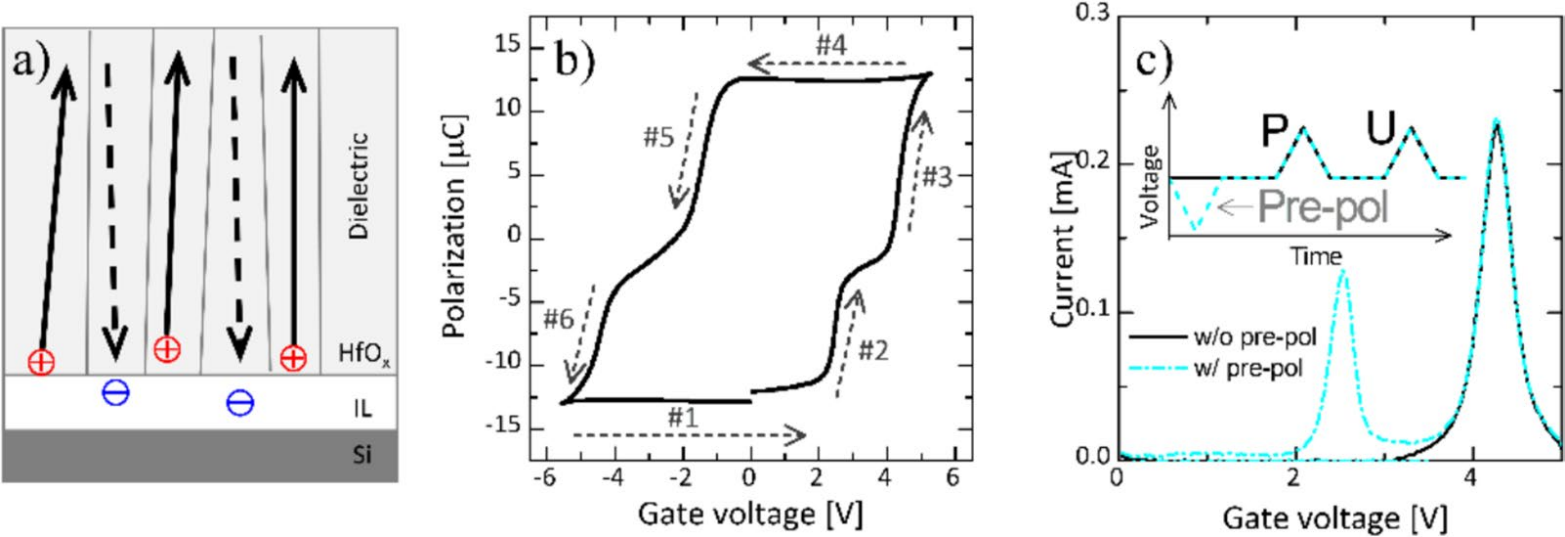
Initial polarization state of ferroelectric material (**a**), polarization characteristic of FTJ before FGA (**b**), and pre-poling effect on positive-up measurement (**c**)

**Fig. 7:**
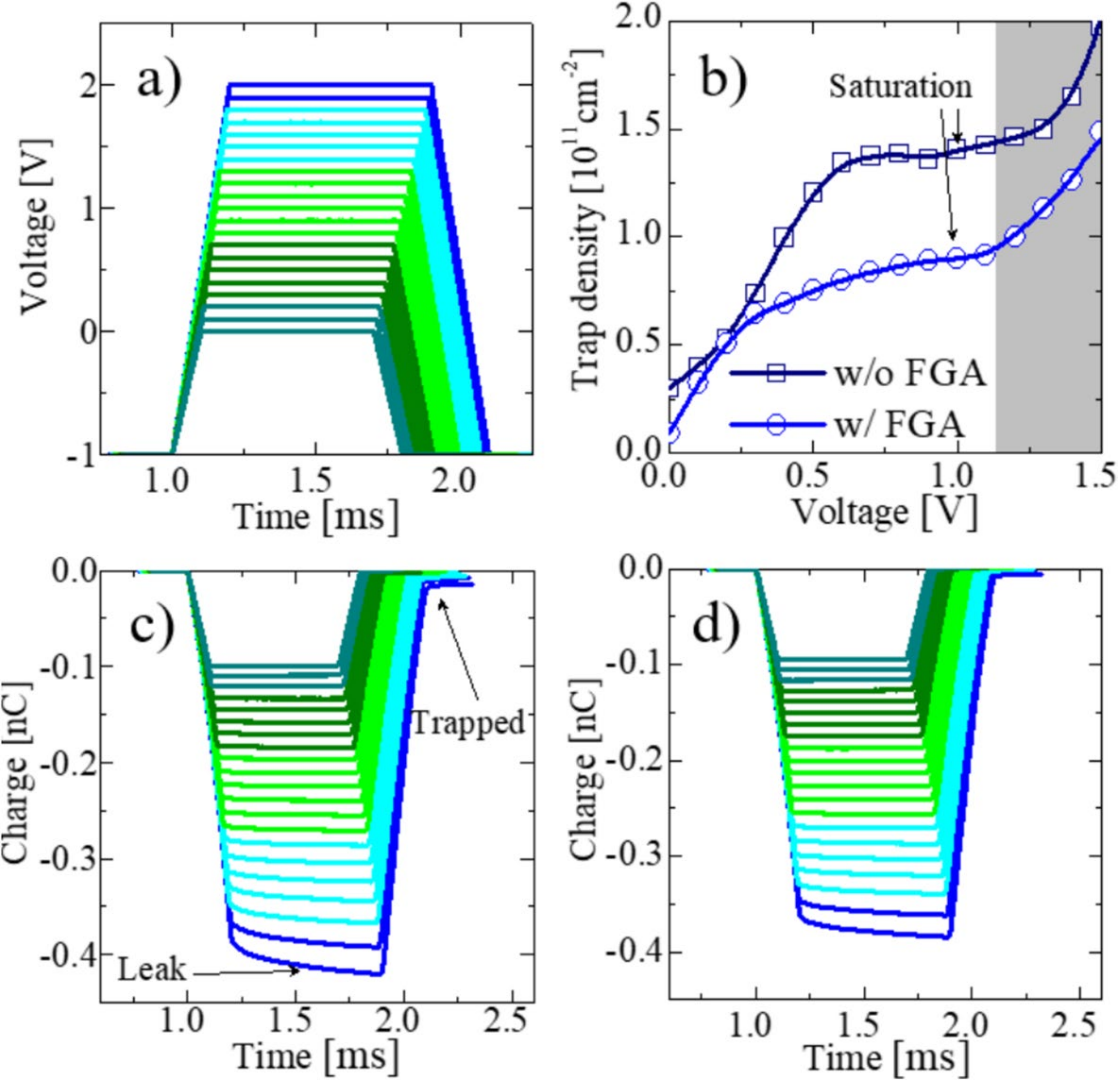
Voltage ramp SPCP applied on FTJ: pulse configuration (**a**), effective trap density (**b**), charge transportation through FTJ without FGA (**c**), and charge transportation through FTJ after FGA (**d**)

**Fig. 8:**
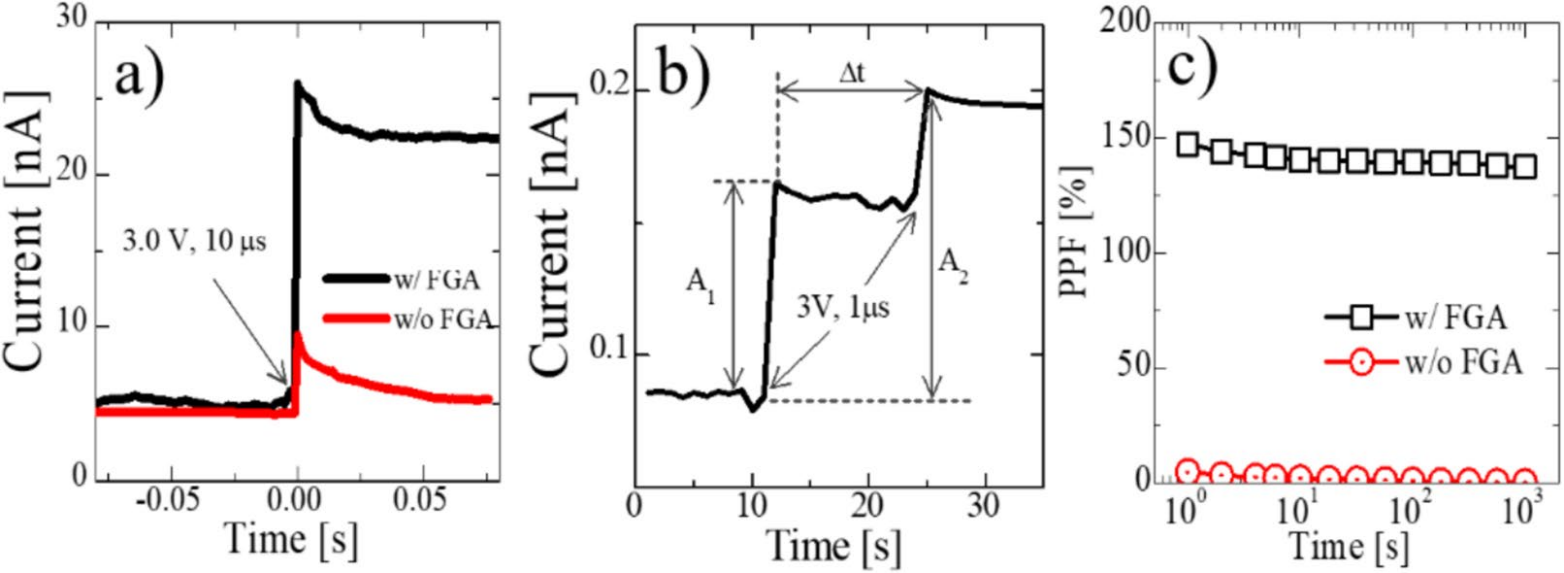
EPSC characteristics of HfO_x_ FTJ triggered by a pulse (3 V, 10 µs) (**a**), a pair of pulses characteristic (**b**), and PPF index as a function of time interval between 2 pulses (**c**)

**Fig. 9:**
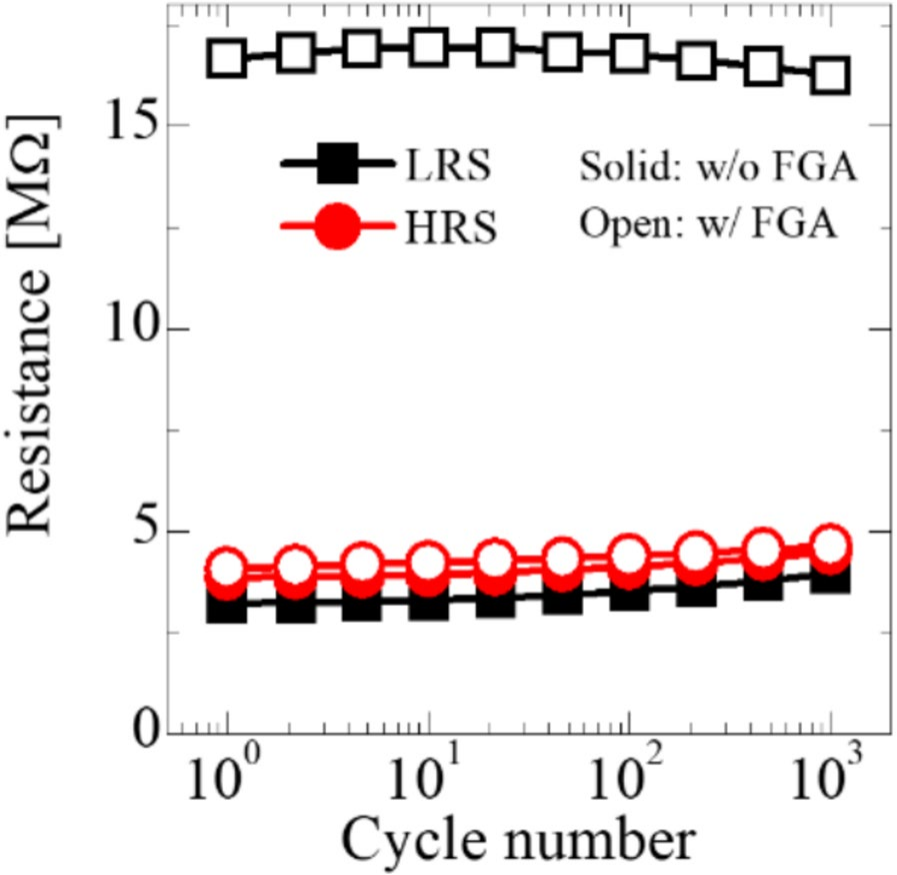
Endurance of the FTJs with and without FGA under a stress condition of 3 V program, −3 V erase, duty cycle 50%, period 2 µs

**Fig. 10:**
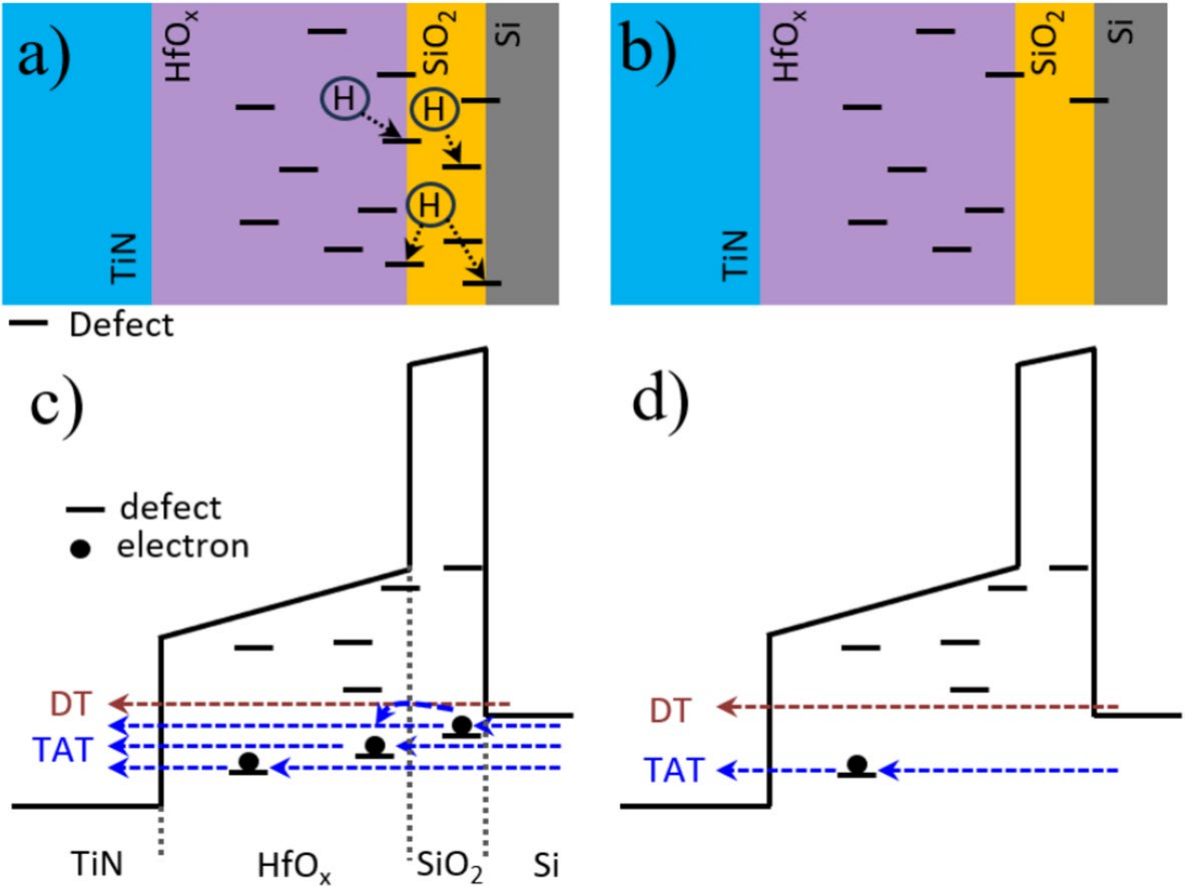
Illustration of dangling bonds passivation in MFIS FTJ before FGA (**a**), after FGA (**b**); illustration of conduction band bending and tunneling currents in MFIS FTJ with low external electric field applied on top electrode: before FGA (**c**) and after FGA (**b**)

**Fig. 11:**
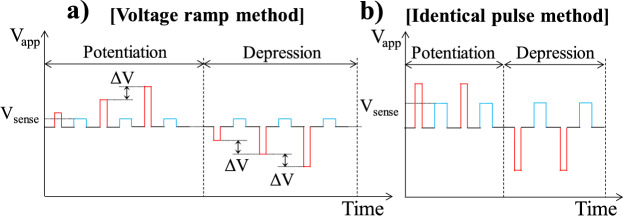
Illustrations pulse schemes for P/D characterization utilizing voltage ramping method (**a**) and identical pulse method (**b**)

**Fig. 12:**
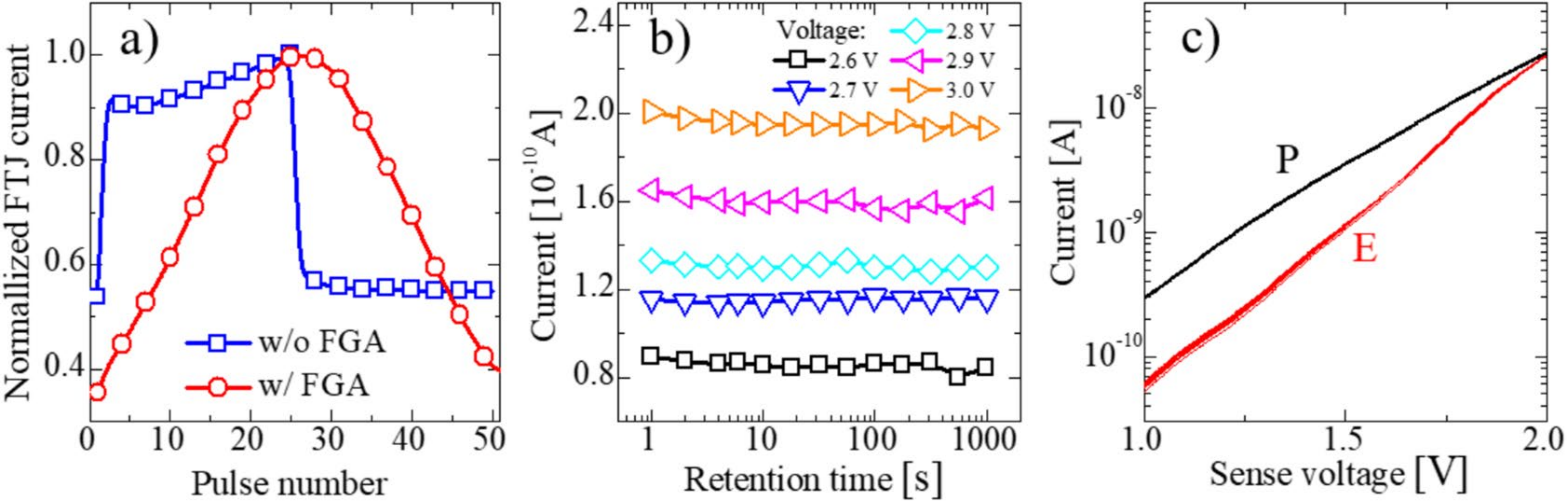
Potentiation and depression characteristic of the FTJ utilizing voltage ramping method (**a**), retention characteristics of the FTJs at several selected states after FGA (**b**), and dependence of LRS and HRS currents at different sense voltages (repeat 20 cycles)

**Fig. 13:**
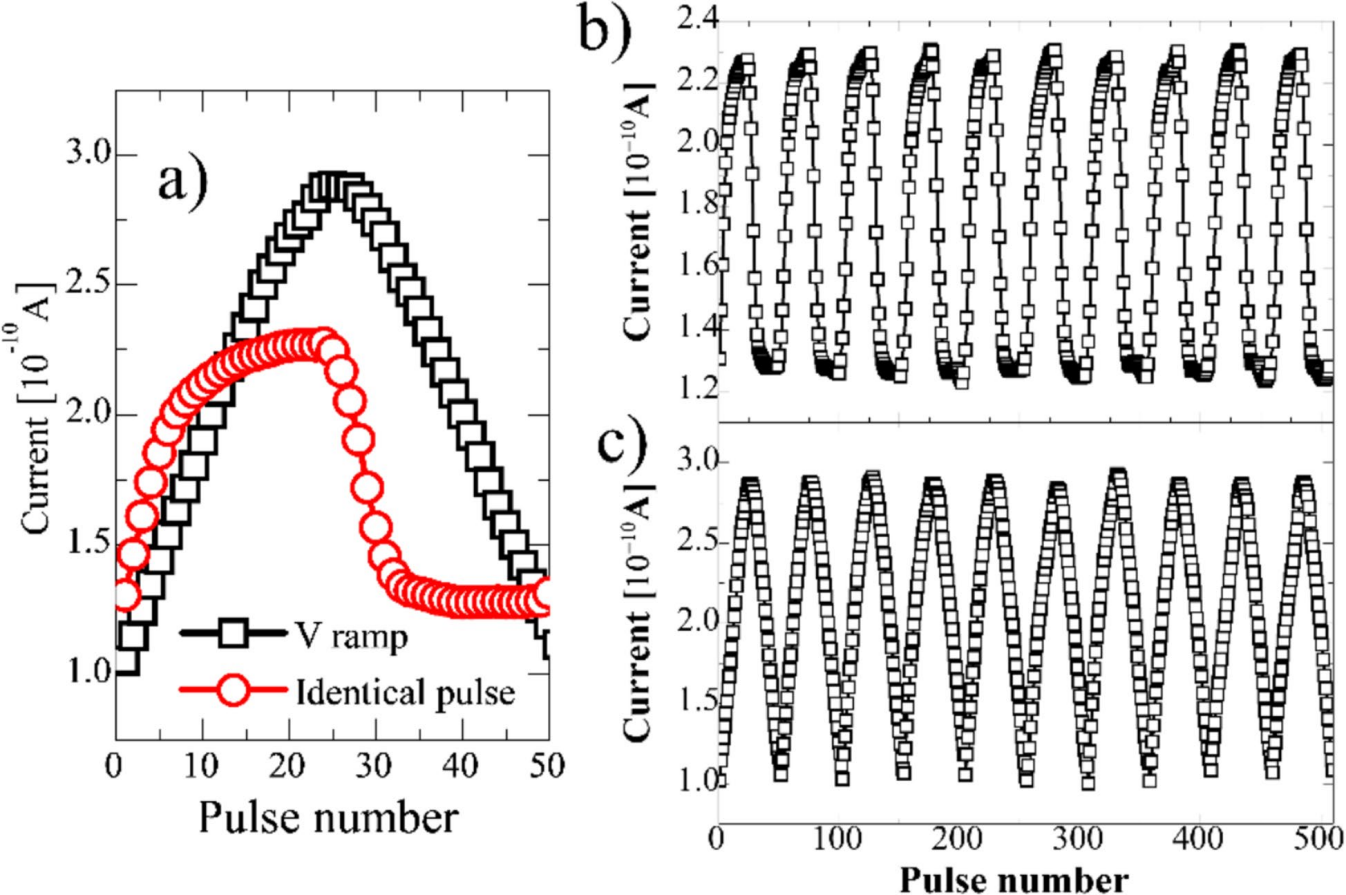
Potentiation and depression (P/D) characteristics of the FTJ after FGA using two different training methods of identical pulse and *V*_app_ ramp (**a**); ten cycles of the P/D of the FTJ characterized by the identical pulse method (**b**) and *V*_app_ ramp method (**c**)

**Fig. 14:**
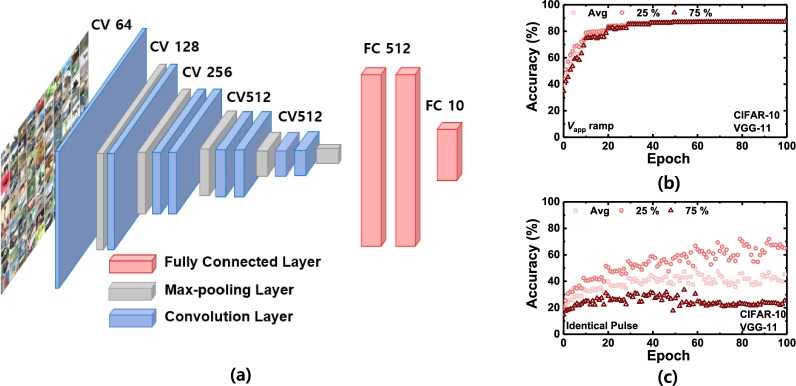
Schematic of VGG-11 network composed of eight convolutional layers, five max-pooling layers, and two fully linked layers (**a**); learning accuracy of the FTJs with voltage ramping method (**b**) and identical pulse scheme (**c**). In each figure, the learning accuracy of the top 25, 75%, and average values of 100 trials are shown

Effects of FGA on the FTJs for neuromorphic applications are investigated using different tools. Figures 4, 5, and 6 summarize the basic characteristics of FTJs. Figure 7 presents defect passivation characterization before and after FGA using the SPCP method. Figures 8, 9, 10, 11, 12 and 13 summarize neuromorphic characteristics of the HfO_x_ FTJs affected by FGA. After obtaining stable P/D characteristics, the applicability of the FGA annealed FTJs as synaptic devices is estimated and summarized in Fig. 14.

Figure 4 shows the effects of the FGA on the double-sweep tunneling current (*I*_tun_) and applied voltage (*V*_app_) characteristics of the FTJ. The forward direction *I*_tun_ (*V*_app_ sweeping from 0 to 3.5 V) was significantly reduced after FGA, whereas the reverse direction *I*_tun_ (from 3.5 to 0 V) was almost unchanged, thereby increasing the MW (Fig. 4a). To verify the origin of the FGA-induced *I*_tun_ change, *I*_tun_/*V*_app_^2^ curves were plotted against 1/*V*_app_ curves (forward direction) before and after FGA to estimate the trap-assisted tunneling (TAT) contribution, because the conducting mechanism of the FTJ can be defined based on the *I*/*V*^2^ versus *1*/*V* curves [22]. In particular, the Fowler–Nordheim (FN) tunneling (FNT) current is a function of voltage as I_FN_ ~ V^2^exp(−1/V), and the TAT could be described as I_TAT_ ~ exp(−1/V). Meanwhile, ln(I/V^2^) in the direct tunneling (DT) region of the metal–insulator-metal or metal–insulator-semiconductor stack gradually decreases as the voltage increases, in contrast to TAT and FN tunneling [22–24]. The tunneling currents are expressed as follows:

2 $${I}_{low}={I}_{DT}+{I}_{TAT}$$

3 $${I}_{high}={I}_{FNT}+{I}_{TAT}$$

where I_low_ and I_high_ denote the tunneling currents under low (below the DT-FNT transition point) and high (above the DT-FNT transition point) electric fields, respectively. The dependence of I_low_ and I_high_ on the electric field depends on the dominant tunneling current. Only the forward sweep data, which is the high-resistance state (HRS), were analyzed to monitor the transition of the conduction mechanism and minimize the effects of the switching current on the tunnel current.

In FTJs with FGA, two primary conduction mechanisms were observed: direct tunneling at low voltages (shaded area in Fig. 4b) and Fowler–Nordheim tunneling (FNT) at high voltages (gray area in Fig. 4b). DT is distinguished from other tunneling mechanisms because ln(I/V^2^) decreases with increasing voltage. The arrow in Fig. 4b denotes the transition voltage from DT (0 to 1.05 V and 0 to −1.4 V) to FNT (1.05 to 4 V and −1.4 to −4 V). The conduction-band offset of the FTJ is shown in the energy-band diagram of the HfO_x_ FTJ (Fig. 4c). Given the negligible charge trapping at low voltages due to the passivation of trap states by the FGA, the tunnel current is governed by DT. In other words, I_TAT_ is negligible in (2). Meanwhile, without FGA, the transition voltages are not observed clearly because TAT (Fig. 4b), rather than DT, is the dominant current-flowing mechanism of the FTJ even at low voltage (approximately up to ±2 V). The absence of transition voltages implies that TAT occurs near the low-voltage region due to severe charge trapping (illustrated in Fig. 4d), and DT. Here, TAT is more dominant than DT.

At high voltage, Fig. 4b shows that TAT and FNT co-exist without FGA, whereas only the FNT is observed for FTJ with FGA, which could be explained by the band structure of FTJ with trap states at high voltage (Fig. 4e). Thus, it can be understood that the *I*_tun_ reduction of the HRS after FGA results from the diminished TAT, which is consistent with a previous report stating that the conduction of HRS in HfO_x_ FTJ is determined by TAT [22]. Additionally, these results are in good agreement with the previous report showing that the traps located near the FE/IL interface are passivated by the inflow of hydrogen during FGA [20], reducing TAT conduction in the FTJs. However, the low-resistance state (LRS) current is almost unchanged after FGA (Fig. 4a). This is evident because Poole–Frenkel emission, which is strongly affected by bulk traps in HfO_x_, is prevalent in the conduction mechanism in the LRS, and the FGA cannot passivate the bulk traps.

Furthermore, the polarization-switching dynamics were investigated for FTJs with and without FGA to clarify the effects of FGA on polarization. Only the polarization-switching current was extracted using the positive-up-negative-down (PUND) measurement method (Fig. 5a) by excluding the transient and leakage current components. It has been reported that the injected hydrogens during FGA prevent the polarization switching by pining the dipole. Decreases in both the polarization switching current and polarization can be observed in Fig. 5a, b, respectively. Figure 5a illustrates two polarization peaks with high intensities at P and N before FGA. However, the first peak at P and second peak at N were significantly reduced, and only a single clear peak appeared after FGA. The missing polarization peaks are expected to be strongly related to the polarization distribution before the polarization reversal is induced by the spatial distribution of oxygen vacancies (positive charge) and defects (negative charge).

Considering the positively shifted P–V curve, the initial polarization state of the FE can be expected (illustrated in Fig. 6a), where the solid and dashed arrows denote the polarization state (P_Bulk_) of the bulk trap states and the polarization state (P_Interface_) of the interface trap states, respectively. When a bias of −5.5 V is applied, and it is swept toward 2 V (#1 in Fig. 6b), P_Bulk_ is polarized up more strongly than P_Interface_ owing to the different polarization direction in the initial state. Thus, P_Interface_ is polarized downward at a lower voltage (#2 in Fig. 6b) than P_Bulk_ (#3 in Fig. 6b). However, at a bias of +5 V (#4 in Fig. 6b), P_Interface_ has a stronger down polarization than P_Bulk_. Accordingly, the polarization reversal of P_Bulk_ occurred first (#5 in Fig. 6b), followed by that of P_Interface_ (#6 in Fig. 6b). This hypothesis can be verified by the change in the switching current based on the pre-poling (Fig. 6c). As depicted in the inset of Fig. 6c, the PU measurements were performed with and without prepoling. In Fig. 6c, the sequential switching currents of P_Interface_ and P_Bulk_ are observed in the positive-bias region with prepoling, whereas only the switching current of P_Bulk_ appears without prepoling. This phenomenon can be understood by the fact that only P_Bulk_ with up polarization can be flipped down by the P pulse because the P_Interface_ exhibits downward polarization in the initial state. However, after FGA, the switching current for the P_Interface_ (Fig. 5a) and the pinched region of the P–V curve (Fig. 5b) disappear because the traps located near the FE/IL interface are passivated by the inflow of hydrogen during FGA. Note that FGA cannot effectively passivate the bulk traps; therefore, the positively shifted P–V curve is maintained even after FGA (Fig. 5b).

Figure 7a shows the configuration of the pulses applied to the top electrode during SPCP characterization. The pulse voltage applied during the SPCP measurement is deliberately set below the ferroelectric switching threshold to ensure that no flat-band voltage shift occurs as a result of ferroelectric domain switching. The dependence of the trap density on the voltage (characterized by the SPCP) is shown in Fig. 7b. The trap density was extracted from the charge-transportation characteristics (Fig. 7c, d). The trap density increases gradually with increasing voltage until saturation near 1 V. A further increase in voltage results in an increase in leakage; therefore, the total charge continued to increase linearly (labeled in Fig. 7c). The trap density is effectively reduced after the FGA (36% reduction). This behavior was reduced after FGA (Fig. 7d) because of the lower trap density and leakage current. Notably, the trap density was extracted at voltages below 1 V, which implies that the interface trap density is effectively passivated (defects above the Fermi level are not detected).

Weight change in the FTJ caused by a short single pulse, excitatory postsynaptic currents (EPSC), is shown in Fig. 8a. Without the FGA, the EPSC is quickly reduced after a pulse, whereas the LRS of the FTJ experiencing the FGA lasts much longer. Weight change characteristic by a paired-pulse (applied to the top electrode) with a delay time between two pulses (Δt) is shown in Fig. 8b. The paired-pulse facilitation (PPF) index was calculated from the current after pulsing A1 and A2 (see Fig. 8b): PPF = (A2 − A1)/A1 × 100%. As expected from the short-term retention shown in Fig. 8a, the PPF index was almost zero on a time scale of more than 1 s (Fig. 8c). In contrast to the FTJ without FGA, the FTJ with FGA shows a PPF index that reduces slowly with time.

Endurance characteristics of the FTJs are shown in Fig. 9 with a stress pulse condition (low voltage −3 V, high voltage 3 V, period 2 μs, and duty cycle 50%). Although the MW of the FTJ was not large, it significantly improved after FGA, indicating that defect passivation using FGA is an effective approach to improve the performance of HfO_x_ FTJ.

The reduction in the trapped density in the FTJ after FGA is the main reason for TAT mitigation; therefore, I_TAT_ in (2) is reduced significantly, and I_DT_ dominates I_low_. Figure 10 illustrates the effect of defect passivation on the tunneling currents when a low electric field is applied to the top electrode. It has been reported that crystallization during RTA results in many defects in MFIS FTJ (Fig. 10a), which contribute to the TAT current. Hence, TAT is considered an important current-flowing mechanism in I_low_ of FTJ with defective films (Fig. 10c). Thermal treatment using FGA is known to be an effective method for passivating dangling bonds, especially near the Si interface, as confirmed by SPCP measurements. Thus, after FGA, the reduction in the interface trap density (shown in Figs. 7b and 10b) mitigates the contribution of I_TAT_ (Fig. 10d) in (2). As a result, the conduction mechanism in the FTJ experiencing FGA is dominated by DT, which can be confirmed by the clear DT-FNT transition point (shown in Fig. 4b). Consequently, owing to trap passivation during FGA, a wider MW (Figs. 4a, 8a, and 9) and improved retention/PPF (Figs. 8a, c, and 9) are achievable by decreasing trapping and detrapping after applying a switching pulse.

The voltage-ramping method shown in Fig. 11a was employed to evaluate the multistate memory operations of the FTJs for neuromorphic device applications. For potentiation (P), the *V*_app_ was ramped from 2.5 to 3.0 V (steps of 0.02 V), whereas the *V*_*a*pp_ was applied from −2.5 to −3.0 V (steps of −0.02 V) for depression (D). The P/D pulse widths were fixed at 1 µs, the rise/fall time was 10 ns, and *I*_tun_ was determined by DC measurement at *V*_G_ = 1 V with an average sampling time of 1 ms to stably monitor low *I*_tun_ (nA order). The delay time between the P/D pulse and sense pulse was fixed at 0.1 s. In the identical pulse method (see Fig. 11b), the potentiation pulse amplitude was fixed at 2.5 V while the depression pulse amplitude was fixed at −2.5 V.

Figure 12a indicates that linear and symmetric P/D characteristics were realized only after FGA, although nonlinear multistate levels (i.e., initial abrupt P/D changes with an increasing number of applied pulses) can be obtained without FGA. This is evident because the trapped charges (electrons) at the defects near the FE/IL interface enhance the polarization switching for potentiation by increasing the electric field (e-field) across the FE, and these trapped charges mostly compensate for the polarization of HfO_x_ to satisfy the charge balance without breakdown of the SiO_2_ IL. In addition, when the trapped charges are detrapped during depression, polarization reversal is accelerated because the polarization cannot be compensated for by the trapped charges. Therefore, the FTJ without FGA undergoes abrupt switching owing to the polarization (polarization reversal) acceleration assisted by charge trapping or detrapping, whereas linear and gradual switching can be secured after FGA because the polarization acceleration is suppressed by the passivation of defects near the FE/IL interface through FGA. Charge-trapping and detrapping at the defects affect the linear/symmetric characteristics but not the TAT. The TAT can be used indirectly to indicate the defect density near the FE/IL interface. Moreover, Fig. 12b shows that the secured linear multilevel memory states remained separate (i.e., they exhibited thermally robust retention characteristics) at 30 °C for 1000 s in a stable manner.

Notably, the MW changed based on the sensing voltage (*V*_sen_), despite performing FGA. This implies that the MW decreases because the HRS increases more rapidly than the LRS with a larger *V*_sen_ (Fig. 12c). This reduction in MW can also be explained by the increase in TAT and FNT at a higher e-field than that of DT. In contrast to the LRS, the HRS is formed by −*V*_app_. This implies that the FE/IL interface traps are likely to be empty because of detrapping, and that TAT can begin to occur at a sufficiently large *V*_sen_. Therefore, a *V*_sen_ of 1 V was selected to minimize *V*_sen_ disturbance.

To estimate the learning performance of FTJs as synaptic devices, the linearity and symmetry of the P/D characteristics in the voltage ramping and identical pulse training methods are compared (Fig. 13a). Figure 13a shows that more optimized linearity and symmetric P/D can be obtained using the voltage ramping method. Ten cycles of P/D (Fig. 13b, c) with stable repeatability and sub-nA current levels were obtained for both methods, which is advantageous for low-power synaptic device applications. The maximum power of the HfO_x_ MFIS FTJ (P) was expected to be approximately 3 µW/cm^2^ based on the following equation:

4 $$P=\frac{{I}_{tun}(LRS)*{V}_{sen}}{Area}.$$

To evaluate the applicability of the FTJs as synaptic devices in CNNs, an eleven-layer visual geometry group (VGG-11) network was simulated using the Canadian Institute for Advanced Research (CIFAR-10) dataset [25]. The VGG-11 network comprises eight convolutional layers, five max-pooling layers, and two fully linked layers, as shown in Fig. 14a. The input to the VGG-11 network is a 32 × 32 pixel RGB image obtained from the CIFAR-10 dataset. The network begins with two sets of convolutional layers, each followed by a Rectified Linear Unit (ReLU) activation function. After each set of convolutional layers, a max-pooling layer is applied to reduce the spatial dimensions of the feature maps. The max pooling operation helps retain important information while reducing the computational complexity. In VGG-11, 2 × 2 max pooling with a stride of 2 is used. Following the convolutional layers, the feature maps are flattened and fed into fully connected layers for classification. VGG-11 contains two fully connected layers, each containing 4096 units. The ReLU activation functions are applied to the fully connected layers. The final layer of the network is a fully connected layer of ten units that correspond to ten classes in the CIFAR-10 dataset.

To verify the influence of the linearity and symmetricity of weights on the learning performance, the pattern recognition accuracy, based on the training method, was simulated in neural networks with 100 trials. Linear weight modulation directly affects the learning performance in on-chip learning owing to uniform Δ*w* values. Figure 14b, c show the learning accuracy of the FTJs with the voltage ramping method and the identical pulse scheme, respectively. In each figure, the learning accuracies of the top 25, 75%, and average values of the 100 trials are shown. The large variation in learning accuracy during identical pulse training results from the nonlinearity of the conductance. In on-chip learning, gradient-based backpropagation is employed, and the FTJs are potentiated or depressed, based on the sign of the gradient. Herein, if over-potentiation or over-depression occurs owing to non-linear conductance updates in a synaptic device, the accuracy at specific epochs is degraded and can therefore fluctuate during training. However, for an incremental pulse scheme, because the conductance can be changed linearly according to the sign of the gradient, a weight can be finely adjusted (updated), and a high accuracy of pattern recognition (up to 90%) can be achieved with a significantly reduced fluctuation as the epoch is repeated. To reflect the behavior of FTJs accurately, P/D characteristics were modeled using experimentally derived parameters. The device conductance modulation was captured through non-linear P/D updates in device properties were included in the simulation. The on-chip learning process incorporates the non-ideal characteristics during weight initialization, forward propagation, backward propagation, and weight updates. These steps were designed to emulate real-world operational conditions, enabling a more realistic assessment of FTJ performance in neuromorphic computing applications. If over-potentiation or over-depression occurs owing to non-linear conductance updates in a synaptic device, the accuracy at specific epochs is degraded and can therefore fluctuate during training. However, for an incremental pulse scheme, because the conductance can be changed linearly according to the sign of the gradient, a weight can be finely adjusted (updated), and a high accuracy of pattern recognition (up to 90%) can be achieved with a significantly reduced fluctuation as the epoch is repeated. Furthermore, the standard deviation is reduced from 4.38 to 0.38 (the mean value of the accuracy variance of the last 10 epochs for all trials). This indicates that Δ*w* remained nearly consistent with the linear weight updates regardless of *w*, and thus, stable learning behaviors can be obtained. However, Δ*w* significantly varies based on *w* with nonlinear weight updates, resulting in significant fluctuation in the recognition accuracy owing to abrupt weight changes.

## Conclusions

In this study, multistate weights were implemented with robust retention, excellent linearity, and symmetric P/D in fabricated HfO_x_ FTJs using FGA. Following FGA, linear and symmetric P/D characteristics were realized with a sub-nA current level and stable repeatability because charge trapping at the interface defects was effectively suppressed, and TAT was significantly mitigated. Based on the increased pattern-recognition accuracy (90%), the HfO_x_ FTJs were found to be suitable for low-power synaptic devices.

## Data Availability

The datasets used and/or analyzed during the current study are available from the corresponding author on reasonable request.

## Abbreviations

FGA: Forming gas annealing
HfO_x_: Hafnium oxide
FTJ: Ferroelectric tunnel junction
TAT: Trap-assisted tunneling
MFIS: Metal-ferrielectric-insulator-semiconductor
MW: Memory window
P/D: Potentiation and depression
MFM: Metal-ferroelectric-metal
FE: Ferroelectric
IL: Insulator
MFIM: Metal-ferroelectric-insulator–metal
HZO: Hafnium zirconium oxide
ALD: Atomic layer deposition
MNIST: Modified National Institute of Standards and Technology
CNN: Convolutional neural network
CIFAR-10: Canadian Institute for Advanced Research
RTA: Rapid thermal annealing
o-phase: Orthorhombic phase
PMA: Post-metal annealing
TEM: Transmission electron microscopy
XRD: X-ray diffraction
PFM: Piezoresponse force microscopy
XPS: X-ray photoelectron spectroscopy
SPCP: Single pulse charge pumping
N_trap_: Trap density
I_SPCP_: Single pulse charge pumping current
V_base_: Pulse based voltage
*Q*_*SPCP*_: Single charge pumping charge
DC: Direct current
I_tun_: Tunneling current
V_app_: Applied voltage
HRS: High resistance
FN: Fowler–Nordheim
FNT: Fowler–Nordheim tunneling
LRS: Low resistance state
PUND: Positive-up-negative-down
EPSC: Excitatory postsynaptic currents
PPF: Paired-pulse facilitation
VGG: Visual geometry group
